# Development of an evidence-based pressure injury prevention care bundle for home-care settings: a systematic review and Delphi study

**DOI:** 10.1186/s12912-026-04694-w

**Published:** 2026-04-27

**Authors:** Shwu-Feng Tsay, Yi-Ting Hu, Shu-Fen Lo

**Affiliations:** 1 Department of Nursing and Health Care, Ministry of Health and Welfare, Taipei, Taiwan; 2 Department of Health Services Administration, College of Public Health, China Medical University, Taichung, Taiwan; 3 Adjunct Professor Rank Technical Expert, School of Nursing, National Taiwan University, Taipei, Taiwan; 4 JBI Taiwan Centre of Holistic Care and Evidence Implementation, Taichung, Taiwan; 5 Department of Nursing, Tzu Chi University, No. 880, Sec. 2, Jianguo Rd, Hualien, 970302 Taiwan

**Keywords:** Pressure injury, Home care nursing, Care bundle, Community-based prevention, Delphi consensus

## Abstract

**Background:**

Pressure injury (PI) is largely preventable yet remains challenging to manage within Taiwan’s home-care system, where daily care is primarily delivered by family carers with intermittent support from visiting nurses. Existing hospital-based PI guidelines often lack contextual relevance for community environments. This study aimed to develop an evidence-based and culturally adapted PI prevention care bundle tailored to home-care practice.

**Methods:**

A three-phase modified e-Delphi process was conducted between August 2023 and August 2024. Phase 1 involved a systematic review to identify relevant evidence sources; Phase 2 comprised an expert workshop with five wound-care specialists; and Phase 3 included two rounds of e-Delphi consultation (21 experts in Round 1 and 20 in Round 2). A total of 8,999 records were identified. Of these, 110 records were screened and 73 full-text reports were assessed for eligibility, resulting in ten evidence sources (comprising eight empirical studies and two clinical guidelines). Item-level consensus was determined using predefined criteria, while Kendall’s coefficient of concordance (W), coefficient of variation (CV), and authority coefficient (Cr) were used to assess panel agreement, rating stability, and expert credibility.

**Results:**

The final care bundle comprised five domains and 28 items adapted from the SSKIN framework. Expert agreement improved from Kendall’s W = 0.321 in Round 1 to 0.388 in Round 2 (both *p* < 0.001). Round 2 CV values ranged from 0.00 to 0.06, and the mean authority coefficient was 0.92, indicating stable consensus and high expert credibility.

**Conclusions:**

The developed care bundle provides a structured, evidence-based framework to support both nurses and family carers in delivering consistent PI prevention in home-care settings. It may also serve as a reference model for regions with similar workforce structures and community-based care systems. Further clinical validation and implementation research are required to assess effectiveness, scalability, and cross-context applicability.

**Supplementary information:**

The online version contains supplementary material available at 10.1186/s12912-026-04694-w.

## Introduction

Pressure injuries (PIs), also referred to as pressure ulcers or bedsores, are localised damage to the skin and underlying soft tissues—most commonly developing over bony prominences—due to sustained pressure, friction, or shear forces [1, 2]. PIs impose significant physiological burdens, including pain, malodour, and infection, as well as psychological distress such as depression and anxiety [3]. In severe cases, they may progress to sepsis, necessitate prolonged hospitalisation, and substantially increase healthcare expenditure [1, 4].

Globally, the incidence of PIs continues to rise in parallel with population ageing and the growing prevalence of chronic diseases [5]. In Taiwan, retrospective analyses from 2001 to 2015 revealed increasing PI trends, with 16.3% of affected individuals requiring surgical debridement, 41.3% experiencing repeated hospitalisation for wound-related complications, and a one-year all-cause mortality rate of 14.3% [6, 7]. Although national cost estimates remain limited, the economic burden is likely substantial and comparable to that reported in the United Kingdom, where annual PI-related care expenditures exceed £3 billion [4]. These findings highlight the urgency of strengthening preventive strategies, particularly within home-care environments where clinical monitoring is less frequent.

PIs are largely preventable through timely and evidence-based interventions and are recognised internationally as indicators of patient safety and quality of care [4, 8]. The care bundle approach—introduced by the Institute for Healthcare Improvement in 2001—combines multiple evidence-based measures into a structured protocol to standardise practice and reduce variation [9, 10]. PI prevention bundles have demonstrated effectiveness in reducing hospital-acquired PIs by up to 90% and improving injury severity and patient outcomes [11–13]. However, most evidence has originated from acute or long-term institutional settings [5, 14, 15], with comparatively limited research addressing community or home-care contexts [16, 17]. Furthermore, existing international guidelines largely presume the availability of professional staff and medical equipment, limiting their relevance to resource-constrained home-care environments.

In Taiwan, home-care nursing services are delivered through intermittent NHI-regulated visits—typically limited to two visits per month for general cases and up to four for individuals with active wounds [18]. As a result, daily PI prevention relies primarily on non-professional family carers, whose knowledge and skill levels vary widely [3, 16]. Consequently, preventive practices may vary and are often experience-based rather than protocol-driven, leading to inconsistent risk assessment and delayed intervention.

Culturally, family members frequently assume the primary caregiving role, whereas home-care nurses’ function mainly as educators, coordinators, and supervisors of care rather than direct providers [8, 19]. Effective PI prevention in home-care settings therefore requires clear, feasible, and family-centred guidance [20]. Developing a contextually adapted framework aligns with Taiwan’s long-term care policies supporting ageing in place and strengthening community-based healthcare support systems [19, 20]. To address this gap, this study adopted a three-phase approach—comprising a systematic review, expert workshop, and two-round e-Delphi consultation—to develop and validate a culturally and contextually relevant PI prevention care bundle tailored to Taiwan’s home-care system. The resulting framework aims to support safe, consistent, and equitable preventive care across varied home environments [21].

## Aim

This study aimed to develop an evidence-based, culturally adapted PI prevention care bundle for Taiwan’s home-care system through a systematic review, expert workshop, and two-round e-Delphi consensus process.

## Methods

### Study design

This study employed a three-phase mixed-methods design comprising: (1) a systematic review, (2) an expert workshop, and (3) a two-round e-Delphi process. This sequential approach enabled the integration of evidence sources and expert consensus to inform the development of a home-care PI prevention care bundle [22]. The study was conducted between August 2023 and August 2024 in accordance with the Guidance on Conducting and Reporting Delphi Studies (CREDES) [23], ensuring methodological rigour, transparency, and reproducibility. By incorporating both evidence sources and clinical expertise, this design supported the development of a contextually relevant and practically feasible bundle for use in home-care settings.

## Phase 1: systematic review

A comprehensive literature search on PI care bundles was conducted across six electronic databases (PubMed, EBSCOhost Nursing Collection, CINAHL, JBI Evidence-Based Practice, the Cochrane Library, and CEPS) and four guideline repositories (NICE; EPUAP/NPIAP/PPPIA; Wounds Australia; and WUWHS). The search combined Medical Subject Headings (MeSH) and free-text terms related to pressure injury, pressure ulcer, care bundle, and community or home care, using Boolean operators and truncation as appropriate.

Evidence sources were eligible for inclusion if they addressed PI prevention in community or home-care settings, evaluated care bundles or key preventive components relevant to bundle development, were published in English or Chinese within the previous ten years, and comprised clinical practice guidelines or relevant primary empirical studies. Sources focusing on mixed wound types, lacking full-text availability, or published as narrative reviews, unpublished manuscripts, conference abstracts, or news articles were excluded.

Two reviewers independently screened titles, abstracts, and full texts, with discrepancies resolved through discussion or consultation with a third reviewer. Reference management was performed using EndNote 20 (Clarivate Analytics), and the final search was completed on 15 August 2023.

The methodological quality of included empirical studies was appraised using appropriate Joanna Briggs Institute (JBI) critical appraisal checklists, while clinical guidelines were evaluated with the AGREE II instrument. Overall evidence certainty was further classified using a modified GRADE approach.

## Phase 2: expert workshop

The expert workshop was conducted with reference to the 2016 and 2019 International Pressure Ulcer/Injury Guidelines [2, 24] and the findings of the preceding systematic review. Five experts with extensive clinical experience in wound management and home-care practice were invited to evaluate the relevance and applicability of the preliminary care bundle components within Taiwan’s home-care context. The workshop was conducted in person and facilitated by the research team to support structured discussion and systematic consensus building. The preliminary framework included 23 domains and 162 care bundle items. During the workshop, experts independently assessed each item for relevance, feasibility, and potential effectiveness in preventing PIs in home-care settings. Items were then retained, revised, or removed based on consensus. Disagreements were resolved through group discussion, or, when necessary, by majority decision. The refined framework generated in this phase served as the foundation for the subsequent modified e-Delphi consensus process.

## Phase 3: modified e-Delphi

### Delphi panel recruitment and selection

Experts were eligible for inclusion if they: (1) held a bachelor’s degree or higher; (2) had at least five years of professional experience in wound care; (3) were currently employed as community nurses, clinical nurses, or physicians; (4) agreed to participate voluntarily; and (5) had completed national or international wound-care training programmes. Exclusion criteria were: (1) inability to complete both Delphi rounds and (2) voluntary withdrawal following full disclosure of study procedures. A total of 30 experts were initially recruited from both urban and rural regions across Taiwan to ensure diverse geographical and clinical representation. Potential participants were identified through professional wound-care associations and home-care service networks and were contacted via email with an information sheet and consent form. The sample size was consistent with established methodological recommendations suggesting that 8–30 participants is sufficient to obtain stable and reliable consensus in Delphi studies [22].

## Modified Delphi process and data collection

Following the expert workshop, purposive sampling was used to recruit panel members, who were invited to participate in the e-Delphi survey via email. Each invitation included an information sheet and informed consent form outlining the study objectives, procedures, and estimated time commitment. Participant confidentiality and anonymity were maintained throughout the process. Two rounds of expert consultation were conducted, each lasting approximately three months [25, 26].

In both rounds, experts rated the importance of each item using a five-point Likert scale ranging from 1 (‘very unimportant’) to 5 (‘very important’) [22]. After each round, participants received anonymised, aggregated feedback summarising the group’s responses and were given the opportunity to revise their ratings in the subsequent round. This controlled, iterative feedback mechanism, together with preserved anonymity, minimised social influence and supported unbiased consensus building.

Consensus was defined as ≥70% agreement and a mean item score ≥ 4.0. Agreement was defined as the proportion of experts rating an item as 4 or 5 on the 5-point Likert scale [22, 25, 27]. The “full-score rate” presented in the results tables refers to the proportion of experts assigning the maximum score of 5 and is reported as a descriptive indicator of rating strength rather than as a criterion for consensus. Stability between rounds was assessed by comparing the direction and magnitude of scoring changes; items demonstrating consistent ratings across rounds were retained, whereas items with persistent disagreement were revised or removed. The timing and applicable stages of each PI-prevention intervention were derived from the 2019 International Pressure Ulcer/Injury Guideline (EPUAP/NPIAP/PPPIA) [2, 24] and subsequently validated through the two Delphi rounds with local experts, ensuring alignment with evidence-based PI progression pathways and home-care practice feasibility.

### Validity, reliability, and rigor

The authority coefficient (Cr) was used to evaluate the credibility of the expert panel and was calculated as Cr = (Ca + Cs) / 2, where Ca represents the basis of judgement (e.g., theoretical analysis, practical experience, or intuitive reasoning), and Cs denotes the expert’s familiarity and confidence regarding the consultation content. Both Ca and Cs were rated on a five-point Likert scale (1 = very unfamiliar/not confident; 5 = very familiar/very confident). The mean Cr value of 0.92 indicated high expert authority and exceeded the accepted threshold of 0.70 [28–30].

The coefficient of variation (CV) was used to assess the stability of expert ratings, with CV ≤ 0.30 considered acceptable and CV < 0.20 indicating strong agreement [30]. Kendall’s coefficient of concordance (W), ranging from 0 to 1, was used to evaluate consensus, with higher values indicating stronger agreement. In this study, the predefined threshold of Kendall’s W ≥ 0.30 was applied exclusively to assess overall panel-level agreement among experts, rather than to individual items or specific domain-level ratings. The predefined methodological quality thresholds—Kendall’s W ≥ 0.30 for overall panel agreement, CV ≤ 0.30 for rating stability, and Cr ≥ 0.70 for expert authority—were applied to evaluate the robustness of the Delphi process [25, 29].

Items that did not meet the predefined item-level consensus criteria were revised based on qualitative feedback and re-assessed in the subsequent round. Anonymised summary statistics and narrative comments were provided after each round to facilitate reflection and convergence. This iterative refinement ensured systematic development of both consensus and non-consensus items [23]. Accordingly, item decisions were based on predefined item-level consensus criteria, while Kendall’s W, CV, and Cr were used exclusively to assess overall agreement, rating stability, and expert authority. No conflicts of interest were identified; therefore, independent Delphi coordination was unnecessary.

### Statistical analysis

All data were entered into Microsoft Excel 2022 (Microsoft Corporation, Redmond, WA, USA) and analysed using IBM SPSS Statistics version 26.0 (IBM Corp., Armonk, NY, USA). Descriptive statistics (frequency, percentage, and mean ± standard deviation) were used to summarise expert ratings. Kendall’s W, together with its chi-square (χ^2^) statistic and corresponding *p*-value, was used to assess overall panel agreement, with *p* < 0.05 indicating statistical significance. A Kendall’s W value ≥ 0.30 was considered indicative of acceptable panel-level agreement. The CV was used to evaluate rating stability, and the Cr was used to assess expert credibility. Agreement was defined as the proportion of experts assigning ratings of 4 or 5 on the five-point Likert scale. Items were considered to have reached consensus when ≥ 70% agreement and a mean score ≥ 4.0 were achieved according to the predefined item-level consensus criteria.

### Ethical considerations

This study was approved by the Institutional Review Board of Hualien Tzu Chi Hospital, Taiwan (IRB No. 112–151-B). Written informed consent was obtained from all participants prior to data collection. Participation was voluntary, with no financial incentives. Personal identifiers were used solely for communication and were removed prior to analysis. All data were anonymised and stored on a password-protected computer accessible only to the research team.

To minimise potential bias, researchers did not influence participant ratings. Consistent with Delphi methodology, all communications were conducted electronically to maintain anonymity and prevent dominance effects. In cases where conflicts of interest were identified, an independent researcher coordinated the Delphi process to ensure transparency and objectivity [23]. Clinical trial number: not applicable.

## Results

### Evidence-based literature review and expert workshop

The database search initially identified 8,999 records. After removing duplicates (*n* = 2,794) and applying a publication date filter to include sources published within the past ten years (*n* = 6,095), 110 records remained for title and abstract screening. Following screening, 37 records were excluded, and 73 reports were sought for retrieval. All reports were successfully retrieved, and 73 full-text reports were assessed for eligibility. After full-text assessment, 63 reports were excluded for predefined reasons, and 10 evidence sources—comprising eight empirical studies and two clinical guidelines—met the inclusion criteria [1, 4, 24, 31–37]. Records excluded during screening were primarily due to population mismatch (e.g. ICU or paediatric settings), inappropriate study design, or lack of relevance to community or home-care pressure injury prevention.

The preliminary framework consisted of 23 domains and 162 care-bundle items. During the expert workshop, these were refined into five core domains aligned with the SSKIN framework—skin assessment, support surfaces, repositioning, incontinence/moisture management, and nutrition [38]—with contextual adaptations to reflect the realities of home-care practice in Taiwan. This process resulted in a preliminary set of 31 care-bundle items that integrated recommendations derived from the included evidence sources with the practical considerations of family-led caregiving and resource variability.

**Fig. 1:**
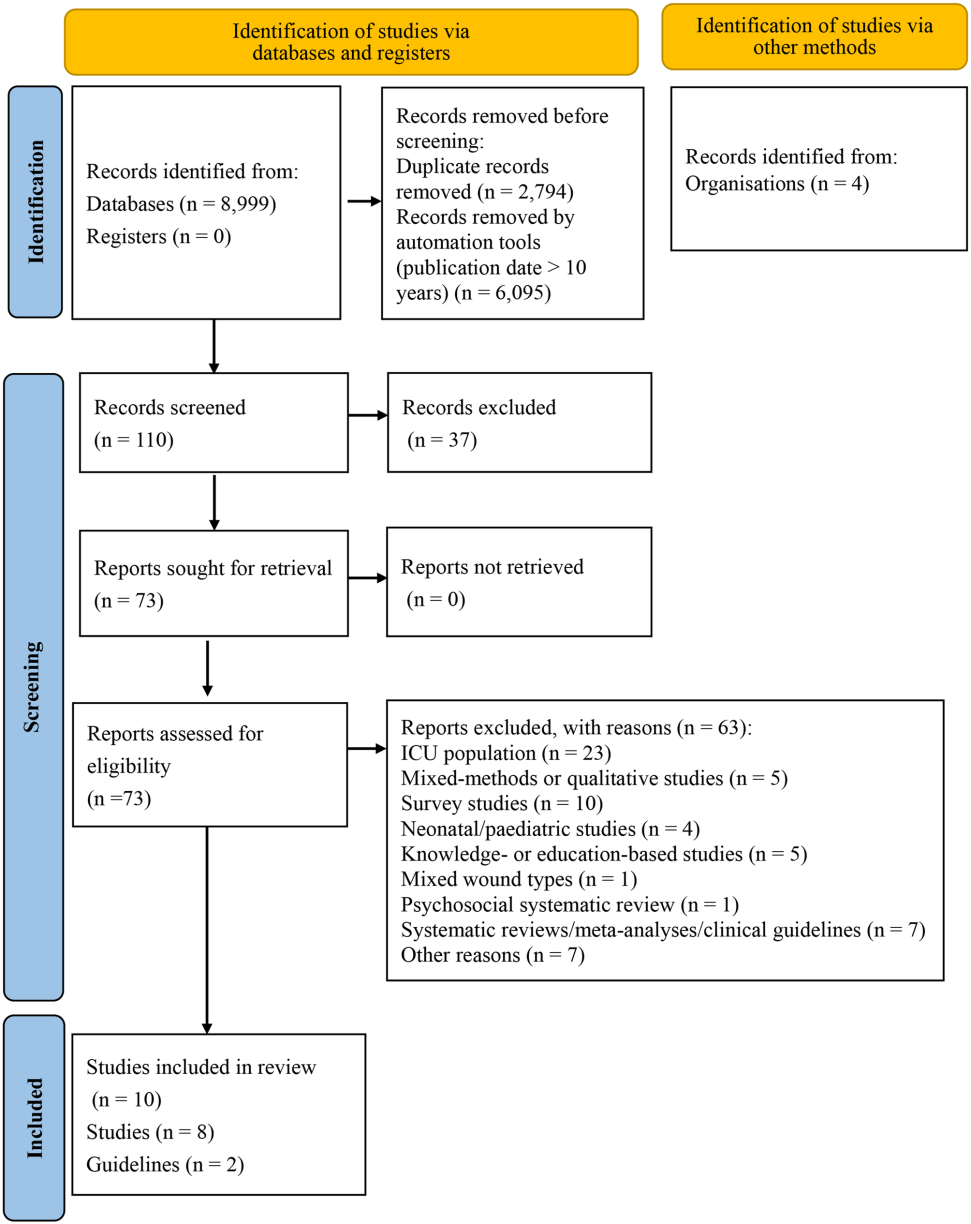
PRISMA 2020 flow diagram illustrating the study selection process

**Fig. 2:**
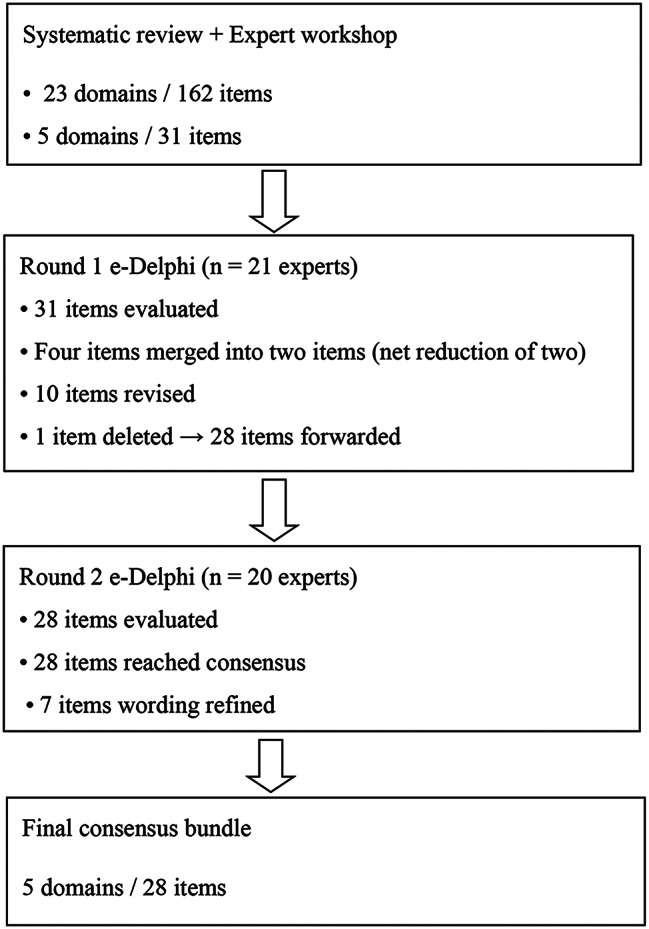
Flowchart of the modified two-round e-Delphi process

Overall, the included evidence sources were of moderate to high methodological quality based on JBI appraisal, with common limitations in sample representativeness and study design. Both clinical guidelines demonstrated high quality across key AGREE II domains, supporting their use as foundational evidence sources. The literature selection process is illustrated in the PRISMA 2020 flow diagram (Fig. 1), and the overall modified e-Delphi procedure is summarised in Fig. 2. The refined framework generated in this phase served as the foundation for the subsequent Delphi consensus rounds.

### Characteristics of experts

A total of 30 professionals from various regions across Taiwan were initially recruited for the Delphi panel between September 2023 and August 2024. Five did not respond and a further five were unable to participate due to scheduling constraints; therefore, 21 experts completed Round 1 and 20 completed Round 2 of the Delphi survey. The panel comprised 12 experts with a master’s degree and nine with a bachelor’s degree. Seventeen participants were home-care nurses, all of whom held national or international wound-care certifications or had completed formal wound-care training programmes.

Overall, 90.5% of the panel were female, with a mean age of 40.6 years (SD = 6.3; range = 31–54). The home-care nurses had an average of 19.6 years of total nursing experience (SD = 6.0; range = 11–34) and 12.8 years of home-care experience (SD = 5.8; range = 5–25). These characteristics indicate that the panel represented a highly experienced and professionally competent group, with diverse clinical backgrounds and geographical distribution across Taiwan. Demographic characteristics for both Delphi rounds are summarised in Table 1.

**Table 1:** Demographic characteristics of Delphi experts (*N* = 21 for Round 1; *N* = 20 for Round 2)

Characteristics	Round 1 (*n* = 21)			Round 2 (*n* = 20)		
	n (%)	Mean (SD)	range	n (%)	Mean (SD)	range
**Age (years)**		40.57(6.29)	31–54		39.90(5.64)	31–54
31–40	14(66.7)			14(66.7)		
41–50	5(23.8)			5(23.8)		
>50	1(9.5)			1(9.5)		
**Years of nursing experience**		19.57(6.03)	11–34		18.95(5.46)	11–34
10–15	6(28.6)			6(30.0)		
16–20	8(38.1)			8(40.0)		
21–25	3(14.3)			2(10.0)		
26–30	2(10.0)			2(10.0)		
31–35	1(5.0)			1(5.0)		
**Years of home care experience**		12.81(5.81)	5–25		12.20(5.22)	5–24
5–9	7(33.3)			7(35.0)		
10–15	7(33.3)			7(35.0)		
16–20	3(14.3)			3(15.0)		
21–25	4(19.0)			3(15.0)		
**Gender**						
Male	2(9.5)			2(10.0)		
Female	19(90.5)			18(90.0)		
**Level of education**						
Undergraduate	9(42.8)			9(45.0)		
Master’s degree	12(57.2)			11(55.0)		
**Nursing job title**						
Home care nurse	17(80.9)			16(80.0)		
Family physician	1(4.7)			1(5.0)		
Community nurse practitioner	2(9.5)			2(10.0)		
Wound care physician	1(4.7)			1(5.0)		
**Region**						
Northern	5(23.8)			5(25.0)		
Central	6(28.6)			5(25.0)		
Southern	5(23.8)			6(25.0)		
Eastern	5(23.8)			4(20.0)		

Abbreviations: SD, standard deviation; %, percentage

## Delphi consultations

### Authority of experts and coordination of expert opinions

In Round 1, the response rate was 100% (*n* = 21), with 15 experts (71.4%) providing written comments. In Round 2, 20 of the 21 experts returned completed surveys (response rate = 95.2%), and 18 experts (90%) provided qualitative feedback. The overall response rate exceeded 90%, indicating strong engagement and sustained participation across both rounds.

The mean Cr of the expert panel was 0.92, surpassing the accepted threshold of 0.70 and demonstrating high professional credibility. Kendall’s W increased from 0.321 in Round 1 to 0.388 in Round 2 (both *p* < 0.001), indicating modestly improved panel convergence across rounds. As Kendall’s W measures inter-expert concordance, these results reflect enhanced alignment and convergence across rounds rather than disagreement. Summary statistics are presented in Table 2.

**Table 2:** Kendall’s W for expert agreement across Delphi rounds

Domain	Items (n)	Kendall’s W	χ^2^	*p*-value
**Round 1 (n = 21)**				
Overall	31	0.321	192.89	<0.001
Skin assessment	7	0.313	43.81	0.002
Support surfaces	5	0.410	40.99	0.004
Repositioning	7	0.350	48.95	<0.001
Incontinence & skin care	6	0.272	32.62	0.037
Nutrition	5	0.646	64.55	<0.001
**Round 2 (n = 20)**				
Overall	28	0.388	209.51	<0.001
Skin assessment	5	0.441	44.10	0.001
Support surfaces	5	0.467	46.66	<0.001
Repositioning	6	0.460	55.23	<0.001
Incontinence & skin care	6	0.297	36.60	0.017
Nutrition	6	0.640	64.16	<0.001

Notes: Kendall’s W ranges from 0 (no agreement) to 1 (complete agreement). A significant *p* < 0.05 indicates agreement beyond chance

### Modifications to indicators

In Round 1, 31 care-bundle items across five domains were evaluated, with mean importance scores ranging from 3.14 to 4.95 and CVs between 0.04 and 0.33. Based on qualitative feedback from the expert panel, four conceptually overlapping items were merged into two consolidated items (net reduction of two items) to reduce redundancy and improve coherence. Ten items were subsequently revised to enhance clarity and contextual applicability, and one item was removed because it did not meet the predefined consensus criteria. Following these modifications, a total of 28 items were retained and circulated to all 21 experts for re-evaluation in Round 2.

### Modifications to specific indicators

During Round 1, the item ‘assess for changes in oedema and consistency with surrounding tissues’ (mean = 3.14; CV = 0.33; full-score rate = 65%) was removed because it did not meet the predefined consensus criteria. In addition, four conceptually overlapping items—‘use fingertip pressure to assess blanching’, ‘minimise pressure on bony prominences during repositioning’, ‘elevate the head of the bed to prevent sliding’, and ‘keep the head of the bed level’—were merged into two consolidated items to reduce redundancy and improve conceptual coherence. Ten further items were revised to enhance clarity and contextual applicability to home-care practice. Following these modifications, 28 items were retained and circulated to the expert panel for Round 2 evaluation. All retained and revised items met the predefined consensus thresholds in Round 2, resulting in a final five-domain, 28-item care bundle (Table 3).

**Table 3:** First-round Delphi results for the PI prevention bundle (*N* = 21)

Domain/Care item	Mean ± SD	CV	Agreement (%)	Full-score rate (%)
**Skin assessment (7 items)**	**4.35 ± 0.21**	**0.05**		
1. Assess skin and tissue for all patients at risk of PI	4.86 ± 0.36	0.07	100.0	95.2
2. Use PI risk assessment tools to evaluate risk of PI (a)	4.38 ± 0.50	0.11	90.0	72.6
3. Assess the skin over all bony prominences for erythema (a)	4.81 ± 0.40	0.08	95.0	89.0
4. Assess skin temperature and soft-tissue consistency (a)	4.81 ± 0.40	0.08	95.0	89.0
5. Reassess if changes in health status increase PI risk	4.62 ± 0.50	0.11	95.0	84.2
6. Assess for changes in edema and consistency (b)	3.14 ± 1.06	0.33	80.0	65.0
7. Use fingertip pressure or transparent board to assess blanching (c)	4.14 ± 0.36	0.09	85.0	70.4
**Support surfaces (5 items)**	**4.85 ± 0.36**	**0.07**		
1. Provide pressure-reducing mattresses for high-risk patients	4.95 ± 0.22	0.04	100.0	95.2
2. Minimize pressure on bony prominences during repositioning	4.76 ± 0.44	0.09	90.0	76.2
3. Avoid donut-shaped devices (a)	4.67 ± 0.58	0.12	85.0	71.4
4. Use prophylactic dressings for heel pressure relief	4.86 ± 0.36	0.07	95.0	85.7
5. Use multi-layer silicone foam dressings (a)	4.81 ± 0.40	0.08	90.0	81.0
**Repositioning (7 items)**	**4.68 ± 0.26**	**0.06**		
1. Reposition patients based on individualized schedule	4.76 ± 0.44	0.09	95.0	85.0
2. Use 30-degree lateral position for at-risk patients	4.43 ± 0.51	0.11	88.0	75.0
3. Elevate the head of bed to prevent sliding (c)	4.90 ± 0.30	0.06	98.0	90.0
4. Use a repositioning sheet to move patients	4.86 ± 0.36	0.07	95.0	86.0
5. Minimize pressure on bony prominences (a)	4.67 ± 0.48	0.10	92.0	81.0
6. Encourage patients to sit in appropriate chair/wheelchair	4.76 ± 0.44	0.09	95.0	86.0
7. Keep the head of the bed as level as possible (c)	4.43 ± 0.51	0.11	85.0	70.0
**Incontinence and skin care (6 items)**	**4.63 ± 0.22**	**0.05**		
1. Ensure skin cleanliness and hydration after urination/defecation	4.76 ± 0.44	0.09	95.0	85.5
2. Use pH-balanced cleanser and protectants	4.43 ± 0.51	0.11	88.0	75.0
3. Avoid vigorous skin rubbing	4.90 ± 0.30	0.06	100.0	95.0
4. Use absorbent diapers for at-risk patients (a)	4.86 ± 0.36	0.07	95.0	85.8
5. Use low-friction textiles	4.67 ± 0.48	0.10	92.0	81.0
6. Use water-based moisturizers	4.76 ± 0.44	0.09	95.0	85.4
**Nutrition (5 items)**	**4.88 ± 0.25**	**0.05**		
1. Assess nutrition and screen for malnutrition	4.90 ± 0.30	0.06	100.0	94.0
2. Encourage adequate fluid intake unless contraindicated (a)	4.90 ± 0.30	0.06	100.0	94.0
3. Provide 30–35 kcal/kg and 1.25–1.5 g/kg protein for malnourished patients (a)	4.90 ± 0.30	0.06	100.0	94.0
4. Provide vitamin/mineral supplements if deficiency suspected (a)	4.71 ± 0.46	0.10	92.0	84.9
5. Reassess nutrition if health status changes	4.95 ± 0.22	0.04	100.0	95.0

Notes**:** Items marked (a) were revised, (b) deleted, and (c) combined in the second Delphi round. Abbreviations**:** Mean ± SD = mean importance score and standard deviation; CV = coefficient of variation; Agreement (%) = proportion of experts rating the item as 4 or 5 on a 5-point Likert scale; Full-score rate (%) = percentage of experts assigning a full importance score (5 on a 5-point Likert scale) to the item; PI = pressure injury

### Final consensus and additional item

Based on panel recommendations to address comorbidities and polypharmacy that may impair nutritional intake in home-care patients, one additional item—“manage symptoms affecting nutritional intake (e.g., pain, nausea)”—was introduced for evaluation in Round 2.

In Round 2, all 28 items met the predefined item-level consensus criteria, with mean importance scores ranging from 4.90 to 5.00, CVs between 0.00 and 0.06, and full-score rates of 94–100%. Seven items were further refined for linguistic precision and contextual clarity based on expert feedback. The newly added nutritional item also met the predefined consensus criteria (mean = 4.90; full-score rate = 94%). Detailed item-level results are presented in Table 4.

**Table 4:** Second-round Delphi results for the home-care PI prevention bundle (*N* = 20)

Domain/Care item	Mean ± SD	CV	Agreement (%)	Full-score rate (%)
**Skin assessment (5 items)**	**4.96 ± 0.08**	**0.02**		
1. Assess skin and tissue for all patients at risk of PI	5.00 ± 0.00	0.00	100.0	100.0
2. Use Braden scale PI risk assessment tools to evaluate the risk of PI (a)	4.95 ± 0.22	0.04	100.0	95.0
3. Assess the skin over all bony prominences for non-blanchable erythema	4.95 ± 0.22	0.04	100.0	95.0
4. Assess skin temperature and tissue consistency changes, especially over bony prominences (a)	4.95 ± 0.22	0.04	100.0	95.0
5. Reassess if changes in the patient’s health status increase their risk of PI	4.95 ± 0.22	0.04	100.0	95.0
**Support surfaces (5 items)**	**4.96 ± 0.08**	**0.02**		
1. Provide pressure-reducing mattresses, such as air cushions, for individuals at high risk of PI	5.00 ± 0.00	0.00	100.0	100.0
2. Minimize pressure on bony prominences during repositioning whenever possible	4.95 ± 0.22	0.05	100.0	95.0
3. Avoid the use of donut-shaped devices at high risk of PI	4.95 ± 0.22	0.05	100.0	95.0
4. Use prophylactic dressings for heel pressure relief and PI prevention	4.95 ± 0.22	0.05	100.0	95.0
5. Use multi-layer silicone foam dressings to protect the skin in at-risk patients	4.95 ± 0.22	0.05	100.0	95.0
**Repositioning (6 items)**	**4.95 ± 0.22**	**0.05**		
1. Reposition patients at risk of or with PI based on an individualized schedule, unless contraindicated	4.95 ± 0.22	0.05	100.0	95.0
2. Use a 30-degree lateral position for individuals at risk of PI	4.95 ± 0.22	0.05	100.0	95.0
3. When the head of the bed is elevated, raise the foot of the bed to prevent sliding downward (a)	4.95 ± 0.22	0.05	100.0	95.0
4. Use a repositioning sheet to move the patient	4.95 ± 0.22	0.05	100.0	95.0
5. Minimize pressure on bony prominences during repositioning and avoid positioning on areas with non-blanchable erythema (a)	4.95 ± 0.22	0.05	100.0	95.0
6. Encourage patients to sit in an appropriate chair or wheelchair, limiting duration	4.95 ± 0.22	0.05	100.0	95.0
**Incontinence and skin care (6 items)**	**4.96 ± 0.07**	**0.01**		
1. Skin care should ensure cleanliness, hydration, and prompt cleaning after urination or defecation	5.00 ± 0.00	0.00	100.0	100.0
2. Use a pH-balanced skin cleanser and select skin protectants to shield the skin from excessive moisture	4.95 ± 0.22	0.05	100.0	95.0
3. Avoid vigorous skin rubbing on bony prominences in individuals at risk of PI	4.95 ± 0.22	0.05	100.0	95.0
4. Use absorbent diapers to prevent moisture damage in incontinent patients	4.95 ± 0.22	0.05	100.0	95.0
5. Use low-friction textiles for patients at risk of PI	4.95 ± 0.22	0.05	100.0	95.0
6. Use water-based moisturizers to hydrate the skin in patients at risk of PI	4.95 ± 0.22	0.05	100.0	95.0
**Nutrition (6 items)**	**4.95 ± 0.10**	**0.02**		
1. Assess nutrition and screen for malnutrition in adults with or at risk of PI	4.95 ± 0.22	0.05	100.0	95.0
2. For patients with PI or at risk, encourage a fluid intake of 30 ml/kg/day, unless contraindicated (e.g., heart failure) (a)	4.95 ± 0.22	0.05	100.0	95.0
3. For adults with PI or at risk, who are malnourished or at risk of malnutrition, administer 30–35 kcal/kg daily and 1.25–1.5 g/kg of protein (a)	4.95 ± 0.22	0.05	100.0	95.0
4. Provide supplements (e.g., vitamins A, C, E; zinc; fatty acids; arginine) if deficiency is confirmed or suspected (a)	4.95 ± 0.22	0.05	100.0	95.0
5. Reassess nutrition if changes in the patient’s health status increase malnutrition risk	5.00 ± 0.00	0.00	100.0	100.0
6. Manage symptoms that affect nutritional intake (e.g., pain, nausea, gastrointestinal issues) and clinical conditions (e.g., dysphagia) (b)	4.90 ± 0.31	0.06	95.0	94.0

Notes: Items marked (a) were revised, (b) added, and (c) combined from the first Delphi round. Abbreviations: Mean ± SD = mean importance score and standard deviation; CV = coefficient of variation; Agreement (%) = proportion of experts rating the item as 4 or 5 on a 5-point Likert scale; Full-score rate (%) = percentage of experts assigning a full importance score (5 on a 5-point Likert scale) to the item; PI = pressure injury

## Discussion

This study adapted the United Kingdom’s SSKIN framework [38, 39] to develop an evidence-based PI prevention care bundle specifically tailored to Taiwan’s home-care context through a systematic review of ten evidence sources, an expert workshop, and a two-round e-Delphi consultation. The final bundle comprises 28 strategies across five domains—skin assessment, support surfaces, repositioning, incontinence and skin care, and nutrition—and provides a clear, standardised framework to guide preventive decision-making. By translating institutional SSKIN principles into simplified, low-resource, and carer-performable strategies, the bundle directly addresses the structural and role-based constraints within Taiwan’s home-care delivery model. In aligning clinical recommendations with the practical realities of family-delivered care, the bundle is designed to enhance feasibility and support more consistent PI prevention practices in resource-limited home environments [37].

### Contextual adaptation and nursing implications

Evidence indicates that care bundles integrating multiple evidence-based interventions can improve care quality, reduce PI incidence, and potentially lower healthcare costs [37, 40]. In Taiwan’s NHI-regulated home-care system, where nursing visits are intermittent [18] and daily care is largely delivered by family carers [20], early risk identification and timely preventive action are particularly important [38]. Within this context, home-care nurses are primarily responsible for clinical assessment, education, supervision, and clinical decision-making, while family carers undertake most routine preventive practices. Monitoring of skin condition and escalation of potential pressure injury risk involve shared responsibilities between nurses and caregivers.

The bundle developed in this study was designed to reflect these structural and role-based characteristics by providing clear, stepwise practices that support caregiver implementation under nurse-led guidance.

Although the SSKIN framework is widely applied in institutional settings [38, 39], its direct application in home care is constrained by limited resources, the central role of family caregiving, and variability in home environments [3]. To address these challenges, the present bundle retained the core principles of SSKIN while incorporating contextual adaptations, including simplified repositioning strategies [38], culturally appropriate nutritional guidance, and the use of affordable low-technology support surfaces [3].

In this model, formal risk assessment is conducted by nurses, while family caregivers observe selected risk-related indicators and report changes to facilitate professional reassessment [18].

These adaptations aim to enhance feasibility and sustainability in resource-limited settings and support more consistent PI prevention practices in home-based care. However, implementation may still face challenges related to caregiver training, resource availability, and adherence monitoring, which warrant further investigation in future studies.

### Risk assessment and preventive practices

Accurate and ongoing risk assessment is fundamental to PI prevention. Although more than 50 validated risk assessment tools exist, the Braden Scale remains the most widely applied internationally and is routinely used in Taiwan’s healthcare system; therefore, it was retained as the primary assessment instrument in this bundle [41]. Early identification of non-blanchable erythema—the earliest visible indicator of tissue damage—may facilitate prompt preventive action and is considered clinically relevant and feasible for family carers in home settings [31, 42].

In addition to risk screening, pressure redistribution is essential for minimising mechanical load. Support surfaces reduce pressure, friction, and shear [43], whereas donut-shaped cushions may increase venous congestion and oedema and should be avoided [1]. Similarly, when elevating the head of the bed for respiratory or cardiovascular comfort, raising the foot of the bed can prevent sliding and shear forces [1, 44]. Repositioning by lifting rather than dragging, careful selection of transfer equipment, and prioritising heel protection further preserve tissue integrity [44]. These principles are reflected in the bundle as clear, actionable strategies appropriate for non-professional carers.

Importantly, the bundle situates these practices within a repeatable surveillance process: risk and skin assessments should be performed at the initial nursing visit and re-evaluated following hospitalisation, acute illness, or any signs of skin deterioration [3]. Embedding this bundle into home-care workflows and piloting it in representative agencies will help to assess feasibility, inform carer training pathways, and generate evidence to guide broader implementation and potential scale-up.

### Nutrition and hydration

Adequate nutritional support is essential for maintaining skin integrity and promoting wound healing; however, malnutrition is frequently under-recognised in home-based care despite its established association with both increased PI incidence and greater injury severity [44, 45]. Symptoms that limit oral intake—such as pain, dysphagia, or nausea—should be identified and managed proactively to prevent nutritional decline [44, 45]. For individuals at risk of PI, recommended intake includes 30–35 kcal/kg/day and 1.25–1.5 g/kg/day of protein, with fluid intake adjusted to approximately 30 ml/kg/day and increased during fever or excessive perspiration [1, 44]. Micronutrients such as arginine, zinc, and vitamins A, C, and E contribute to collagen synthesis and tissue repair, and were therefore incorporated into the bundle under professional supervision [34, 44].

By embedding these recommendations within a structured home-care framework, the bundle emphasises practical strategies that family carers can apply consistently, helping to reduce the risk that nutritional needs are overlooked in daily preventive care. This approach aligns evidence-based nutritional principles with the realities of home-based caregiving, enhancing feasibility and supporting more consistent nutritional care practices related to wound healing.

### End-of-life and comfort care

As demand for home-based hospice care continues to increase in Taiwan, PI prevention must be aligned with patient-centred end-of-life goals [46]. In this context, repositioning is no longer solely a preventive technique but becomes part of comfort-focused decision-making. The frequency and method of repositioning should therefore be individualised according to patient tolerance, comfort preferences, and overall clinical condition, with family carers involved in shared decision-making to balance potential benefits against physical burden [46, 47]. This person-centred approach reframes PI prevention as a means of preserving dignity and minimising suffering, rather than as an intervention pursued for its own sake, and reflects the bundle’s emphasis on compassionate, evidence-based care in home settings.

To support implementation, the bundle is intended to be integrated into routine home-care workflows and piloted within representative care agencies to assess feasibility and acceptability. Further research is required to evaluate its effects on PI incidence, carer burden, and healthcare utilisation across diverse communities, particularly in rural or resource-limited settings. Such evidence may guide ongoing refinement of the bundle and inform policy development related to home-care accreditation, carer training, and quality assurance under Taiwan’s long-term care framework.

## Limitations

This study has several limitations. First, the Delphi panel was relatively small (*n* = 20) and recruited using purposive sampling, which may limit the generalisability of the findings. Second, the panel was predominantly female and largely composed of home-care nurses, which may introduce disciplinary or gender-related perspective bias. Third, the moderate Kendall’s W values (0.321–0.388) indicate some variability in expert agreement; however, such levels are commonly observed in Delphi studies addressing complex multidisciplinary issues. Fourth, the systematic review included only English- and Chinese-language publications, and the number of included evidence sources was limited, which may have constrained the breadth of evidence informing bundle development.In addition, the Delphi questionnaire was not formally piloted prior to administration, which may have influenced item interpretation despite iterative refinement during the Delphi rounds. Furthermore, this study focused on bundle development rather than clinical effectiveness; therefore, external validation and field testing in routine home-care settings are required to assess applicability and implementation feasibility. Future research should involve broader and more diverse expert recruitment and multicentre implementation studies to evaluate feasibility, sustainability and effectiveness in real-world home-care contexts.

## Conclusions

This study developed a five-domain, 28-item evidence-based care bundle for preventing pressure injuries in Taiwan’s home-care settings through a systematic review and a two-round e-Delphi consultation with wound-care and home-care experts. The bundle provides a standardised framework to support both nurses and family carers in delivering consistent, proactive, and evidence-informed preventive care. Although developed within Taiwan’s healthcare context, the framework has potential transferability to other settings characterised by workforce limitations and reliance on informal caregiving.

By emphasising simple, low-cost, and feasible strategies—such as routine skin inspection, repositioning, use of appropriate support surfaces, incontinence management, and nutritional support—the bundle is designed to be implemented with minimal equipment and training, making it particularly suitable for resource-limited or community-based environments. Future research should prioritise clinical validation, feasibility testing within routine home-care workflows, and cross-national adaptation to assess its effectiveness and scalability. In addition, multicentre implementation trials and longitudinal outcome evaluations are needed to determine the bundle’s real-world impact and inform system-level integration. Such work may inform the development of future community-based pressure injury prevention strategies and further strengthen the leadership role of nurses in advancing evidence-based home-care practice.

## Electronic supplementary material

Below is the link to the electronic supplementary material.

Supplementary Material 1

## Data Availability

The datasets generated and/or analysed during the current study contain potentially identifiable participant information and are therefore not publicly available. Data are available from the corresponding author on reasonable request.

## References

[1] Gould LJ, Alderden J, Aslam R, Barbul A, Bogie KM, El Masry M, et al. WHS guidelines for the treatment of pressure ulcers—2023 update. Wound Repair Regener. 2024;32(1):6–33. 10.1111/wrr.13130. 10.1111/wrr.13130 PMC11403384 37970711

[2] Haesler E, Kottner J, Cuddigan J. The 2014 international pressure ulcer guideline: methods and development. J Adv Nurs. 2017;73(6):1515–30. 10.1111/jan.13241. 27987246 10.1111/jan.13241

[3] Lo SF, Chuang ST, Liao PL. Challenges and dilemmas of providing healthcare to patients in home care settings with hard-to-heal wounds [Chinese]. J Nurs. 2021;68(4):89–95. 10.6224/JN.202108_68(4).11 34337707

[4] Lavallée JF, Gray TA, Dumville J, Cullum N. Preventing pressure ulcers in nursing homes using a care bundle: a feasibility study. Health Soc Care Community. 2019;27(4):e417–27. 10.1111/hsc.12742. 10.1111/hsc.12742 PMC6618244 30919525

[5] Aloweni F, Lim SH, Gunasegaran N, Ostbye T, Ang SY, Siow KCE. Community-acquired pressure injuries: prevalence, risk factors and effect of care bundles—an integrative review. J Clin Nurs. 2024, In press.33(12):4618–34. 10.1111/jocn.17431. 39370544 10.1111/jocn.17431

[6] Tai CH, Wang JH, Lo SF, Tsay SF, Yang CC, Oy AC, et al. Incidence, prevalence, and medical costs of pressure injuries in Taiwan from 2001 to 2015: results of a retrospective cohort study. J Clin Nurs. 2025, In press.34(4):1264–76. 10.1111/jocn.17149. 38629347 10.1111/jocn.17149

[7] Lo ZJ, Lim X, Eng D, Car J, Hong Q, Yong E, et al. Clinical and economic burden of wound care in the tropics: a 5-year institutional population health review. Int Wound J. 2020;17(3):790–803. 10.1111/iwj.13333. 32149471 10.1111/iwj.13333 PMC7948834

[8] Ministry of Health and Welfare (Taiwan). Operational guidelines for long-term care professional services-appendix II: guidelines for home nursing instruction and consultation services [Internet]. 2024. Available from: https://1966.gov.tw/LTC/cp-6451-70123-207.html. 2025 May 30.

[9] Demir AS, Karadağ A. Impact of care bundles prevention of hospital-acquired pressure injuries: a systematic review and meta-analysis. Nurs Open. 2025;12(3):e70173. 10.1002/nop2.70173. 10.1002/nop2.70173 PMC11906361 40083077

[10] Resar RG, Griffin FA, Haraden C, Nolan TW. Using care bundles to improve healthcare quality. Cambridge (MA): Institute for Healthcare Improvement; 2012.

[11] Kandula UR. Impact of multifaceted interventions on pressure injury prevention: a systematic review. BMC Nurs. 2025;24(1):11. 10.1186/s12912-024-02558-9. 39762920 10.1186/s12912-024-02558-9 PMC11702047

[12] Altaş G, Çelik S. Evaluation of a pressure injury prevention care bundle in an ICU in Turkey. Adv Skin Wound Care. 2023;36(12):658–65. 10.1097/ASW.0000000000000070. 37983579 10.1097/ASW.0000000000000070

[13] Zhang X, Wu Z, Zhao B, Zhang Q, Li Z. Implementing a pressure injury care bundle in Chinese intensive care units. RMHP. 2021;14:2435–42. 10.2147/RMHP.S292579. 10.2147/RMHP.S292579 PMC8187104 34113197

[14] Rivera J, Donohoe E, Deady-Rooney M, Douglas M, Samaniego N. Implementing a pressure injury prevention bundle to decrease hospital-acquired pressure injuries in an adult critical care unit: an evidence-based, pilot initiative. Wound Manag Prev. 2020;66(10):20–28. 10.25270/wmp.2020.10.2028. 33048828

[15] Roberts S, Wallis M, McInnes E, Bucknall T, Banks M, Ball L, et al. Patients’ perceptions of a pressure ulcer prevention care bundle in hospital: a qualitative descriptive study to guide evidence-based practice. Worldviews Ev Based Nurs. 2017;14(5):385–93. 10.1111/wvn.12226. 10.1111/wvn.12226 28395394

[16] Lin HC, Chen KM, Lee BO, Wu CF, Yu CY, Wang HH. Challenges and prospects of home wound care in Taiwan. Chia-Chi Nurs. 2024;24(1):34–42.

[17] Sugathapala R, Latimer S, Balasuriya A, Chaboyer W, Thalib L, Gillespie BM. Prevalence and incidence of pressure injuries among older people living in nursing homes: a systematic review and meta-analysis. Int J Multiling Nurs Stud. 2023;148:104605. 10.1016/j.ijnurstu.2023.104605. 10.1016/j.ijnurstu.2023.104605 37801939

[18] National Health Insurance Administration (Taiwan). Medical service payment items-home nursing visit (code 05332C) [Internet]. 2024. Available from: https://www.nhi.gov.tw. 2025 May 30.

[19] Liu YC, Chen CF. A historical overview of policy perspectives towards informal care in Taiwan (1996-2023). Health Policy (New Y). 2025;152:105239. 10.1016/j.healthpol.2024.105239. 10.1016/j.healthpol.2024.105239 39837056

[20] Tsao WY, Yeh LY, Chen YJ, Hung HC, Hsu MY. Home care information needs of caregivers of patients with pressure injury. J Welf Technol Serv Manag. 2019;7(1):24–33.

[21] Chen CF, Fu TH. Policies and transformation of long-term care system in Taiwan. Ann Geriatr Med Res. 2020;24(3):187–94. 10.4235/agmr.20.0038. 33012140 10.4235/agmr.20.0038 PMC7533198

[22] Shang Z. Use of Delphi in health sciences research: a narrative review. Med (Baltim). 2023;102(7):e32829. 10.1097/MD.0000000000032829. 10.1097/MD.0000000000032829 PMC9936053 36800594

[23] Jünger S, Payne SA, Brine J, Radbruch L, Brearley SG. Guidance on conducting and REporting DElphi studies (CREDES) in palliative care: recommendations based on a methodological systematic review. Palliat Med. 2017;31(8):684–706. 10.1177/0269216317690685. 28190381 10.1177/0269216317690685

[24] Kottner J, Cuddigan J, Carville K, Balzer K, Berlowitz D, Law S, et al. Prevention and treatment of pressure ulcers/injuries: the protocol for the second update of the international clinical practice guideline 2019. J Tissue Viability. 2019;28(2):51–58. 10.1016/j.jtv.2019.01.001. 30658878 10.1016/j.jtv.2019.01.001

[25] Schifano J, Niederberger M. How Delphi studies in the health sciences find consensus: a scoping review. Syst Rev. 2025;14(1):14. 10.1186/s13643-024-02738-3. 39810238 10.1186/s13643-024-02738-3 PMC11734368

[26] Spranger J, Homberg A, Sonnberger M, Niederberger M. Reporting guidelines for Delphi techniques in health sciences: a methodological review. Z für Evidenz Fortbild und Qualität im Gesundheitswes. 2022;172:1–11. 10.1016/j.zefq.2022.04.025. 10.1016/j.zefq.2022.04.025 35718726

[27] Wang Z, Fan J, Chen L, Xie L, Huang L, Ruan Y, et al. Strategies to preventing pressure injuries among intensive care unit patients mechanically ventilated in prone position: a systematic review and a Delphi study. Front Med. 2023;10:1131270. 10.3389/fmed.2023.1131270. 10.3389/fmed.2023.1131270 PMC10461099 37644983

[28] Hong G, Liu D, Zhao Y, Zhai Y, Zhao F, Wang Y, et al. Establishment of the benchmarking tool for evaluating the operation of biorepositories for pathogenic resource using a modified Delphi method. Biosaf Health. 2024;6(4):199–205. 10.1016/j.bsheal.2024.05.001. 40078664 10.1016/j.bsheal.2024.05.001 PMC11894954

[29] Nasa P, Jain R, Juneja D. Delphi methodology in healthcare research: how to decide its appropriateness. WJM. 2021;11(4):116–29. 10.5662/wjm.v11.i4.116. 34322364 10.5662/wjm.v11.i4.116 PMC8299905

[30] Wang Y, Song X, Wang S, Bai T, Li R, Liu H, et al. Construction of pain management strategies after Hepatectomy: evidence summary and Delphi study. JPR. 2024;17:4541–59. 10.2147/JPR.S494243. 10.2147/JPR.S494243 PMC11687292 39742353

[31] Edsberg LE, Black JM, Goldberg M, McNichol L, Moore L, Sieggreen M. Revised national pressure ulcer advisory panel pressure injury staging system. J Wound Ostomy Cont Nurs. 2016;43(6):585–97. 10.1097/WON.0000000000000281. 10.1097/WON.0000000000000281 PMC5098472 27749790

[32] Hahnel E, El Genedy M, Tomova-Simitchieva T, Hauß A, Stroux A, Lechner A, et al. The effectiveness of two silicone dressings for sacral and heel pressure ulcer prevention compared with no dressings in high-risk intensive care unit patients: a randomized controlled parallel-group trial. Br J Dermatol. 2020;183(2):256–64. 10.1111/bjd.18621. 31628863 10.1111/bjd.18621

[33] Johal J. Evidence summary: pressure injury prevention-skin care. JBI EBP Database. 2023;JBI-ES-541–544.

[34] Langer G, Wan CS, Fink A, Schwingshackl L, Schoberer D. Nutritional interventions for preventing and treating pressure ulcers. Cochrane Database Systematic Rev. 2024;2024(2): CD003216. 10.1002/14651858.CD003216.pub3. 10.1002/14651858.CD003216.pub3 PMC10860148 38345088

[35] Munoz N, Posthauer ME, Cereda E, Schols J, Haesler E. The role of nutrition for pressure injury prevention and healing: the 2019 international clinical practice guideline recommendations. Adv Skin Wound Care. 2020;33(3):123–36. 10.1097/01.ASW.0000653144.90739.ad. 32058438 10.1097/01.ASW.0000653144.90739.ad

[36] Martin S, Holloway S. Pressure ulcers: a clinical audit to determine compliance against the aSsking framework in an adult community nursing setting. Br J Community Nurs. 2024;29(Suppl Sup9):S28–34. 10.12968/bjcn.2024.0064. 10.12968/bjcn.2024.0064 39240816

[37] Chaboyer W, Latimer S, Priyadarshani U, Harbeck E, Patton D, Sim J, et al. The effect of pressure injury prevention care bundles on pressure injuries in hospital patients: a complex intervention systematic review and meta-analysis. Int J Multiling Nurs Stud. 2024;155:104768. 10.1016/j.ijnurstu.2024.104768. 10.1016/j.ijnurstu.2024.104768 38642429

[38] McCoulough S. Adapting a SSKIN bundle for carers to aid identification of pressure damage and ulcer risks in the community. Br J Community Nurs. 2016;21(Sup6):S19–25. 10.12968/bjcn.2016.21.Sup6.S19. 10.12968/bjcn.2016.21.Sup6.S19 27297573

[39] Whitlock J. SSKIN bundle: preventing pressure damage across the health-care community. Br J Community Nurs. 2013;18(Sup9):S32–9. 10.12968/bjcn.2013.18.Sup9.S32. 10.12968/bjcn.2013.18.sup9.s32 24575601

[40] Yilmazer T, Tuzer H. The effect of a pressure ulcer prevention care bundle on nursing workload costs. J Tissue Viability. 2022;31(3):459–64. 10.1016/j.jtv.2022.05.004. 35595597 10.1016/j.jtv.2022.05.004

[41] Huang C, Ma Y, Wang C, Jiang M, Yuet Foon L, Lv L, et al. Predictive validity of the braden scale for pressure injury risk assessment in adults: a systematic review and meta-analysis. Nurs Open. 2021;8(5):2194–207. 10.1002/nop2.792. 33630407 10.1002/nop2.792 PMC8363405

[42] Shi C, Dumville JC, Cullum N. Skin status for predicting pressure ulcer development: a systematic review and meta-analyses. Int J Multiling Nurs Stud. 2018;87:14–25. 10.1016/j.ijnurstu.2018.07.003. 10.1016/j.ijnurstu.2018.07.003 30015089

[43] Kohta M, Ohura T, Tsukada K, Nakamura Y, Sukegawa M, Kumagai E, et al. Inter-rater reliability of a pressure injury risk assessment scale for home care: a multicenter cross-sectional study. JMDH. 2020;13:2031–41. 10.2147/JMDH.S291162. 33376343 10.2147/JMDH.S291162 PMC7765679

[44] Haesler E. Prevention and treatment of pressure ulcers/injuries:clinical practice guideline. NPIAP/EPUAP/PPPIA; 2025.

[45] Haavisto E, Stolt M, Puukka P, Korhonen T, Kielo-Viljamaa E. Consistent practices in pressure ulcer prevention based on international care guidelines: a cross-sectional study. Int Wound J. 2022;19(5):1141–57. 10.1111/iwj.13710. 34761513 10.1111/iwj.13710 PMC9284652

[46] Lo SF, Wu LY, Lo YC. [Management of patients with Kennedy terminal ulcer: challenges and best practices]. Hu Li Za Zhi. 2023;70(2):95–101. 10.6224/JN.202304_70(2).12. 36942547 10.6224/JN.202304_70(2).12

[47] Latimer S, Shaw J, Hunt T, Mackrell K, Gillespie BM. Kennedy terminal ulcers: a scoping review. J Hosp Palliat Nurs. 2019;21(4):257–63. 10.1097/NJH.0000000000000563. 30933013 10.1097/NJH.0000000000000563

