# Supplementary material for: Development of an evidence-based pressure injury prevention care bundle for home-care settings: a systematic review and Delphi study
Source: BMC Nurs. 2026 Apr 27;25:523. doi: 10.1186/s12912-026-04694-w (PMC13251043; doi:10.1186/s12912-026-04694-w)
Supplement: Supplementary file 1 — Supplementary Material 1 [file 12912_2026_4694_MOESM1_ESM.docx]

Supplementary Table S1. Mapping of included evidence to care-bundle domains and item formulation

| Author(s), Year | Evidence type | Care setting | Supported domain(s) | Role in care-bundle development |
| --- | --- | --- | --- | --- |
| Gould et al., 2024 | International clinical practice guideline | Multisetting | Risk/skin assessment; Repositioning; Support surfaces; Nutrition; Skin care | Informed refinement of domain content and alignment with updated international best-practice recommendations |
| Lavallée et al., 2019 | Feasibility study (care bundle) | Nursing home | Risk assessment; Repositioning; Skin care; Education | Informed feasibility, acceptability, and implementation considerations relevant to care-bundle development |
| Kottner et al., 2019 | International guideline protocol | Multisetting | Risk assessment; Skin assessment; Prevention framework | Provided methodological and conceptual guidance for evidence-based domain selection and prevention frameworks |
| Edsberg et al., 2016 | International clinical practice guideline | Acute, long-term, and community care | Risk/skin assessment; Repositioning; Support surfaces; Nutrition; Skin care | Provided foundational international recommendations and informed initial identification of core preventive domains |
| Hahnel et al., 2020 | Randomised controlled trial | High-risk adult patients | Support surfaces; Prophylactic skin protection | Provided empirical evidence supporting inclusion of support surface use and prophylactic dressing components |
| Johal, 2023 | JBI Evidence summary | Multisetting | Skin care; Incontinence-associated skin care | Synthesised best-practice evidence informing skin care, moisture management, and incontinence-related preventive items |
| Langer et al., 2024 | Systematic review | Adult care | Nutrition; Hydration | Supported inclusion of nutrition and hydration as core preventive domains based on synthesised high-level evidence |
| Muñoz et al., 2020 | International clinical practice guideline | Multisetting | Nutrition; Hydration | Informed nutrition- and hydration-related preventive domains relevant toPI prevention |
| Martin & Holloway, 2024 | Observational study | Community and home care | Risk assessment; Repositioning; Skin care | Provided contextual evidence supporting applicability of preventive domains in community and home-care settings |
| Chaboyer et al., 2024 | Systematic review and meta-analysis | Hospital and transitional care | Risk assessment; Repositioning; Skin care; Education | Supported the rationale for bundled preventive approaches and informed selection of key bundle components |
